# Muscle RING Finger-1 Promotes a Maladaptive Phenotype in Chronic Hypoxia-Induced Right Ventricular Remodeling

**DOI:** 10.1371/journal.pone.0097084

**Published:** 2014-05-08

**Authors:** Matthew J. Campen, Michael L. Paffett, E. Sage Colombo, Selita N. Lucas, Tamara Anderson, Monique Nysus, Jeffrey P. Norenberg, Ben Gershman, Jacob Hesterman, Jack Hoppin, Monte Willis

**Affiliations:** 1 Department of Pharmaceutical Sciences, College of Pharmacy, University of New Mexico Health Sciences Center, Albuquerque, New Mexico, United States of America; 2 Radiopharmaceutical Sciences Program, College of Pharmacy, University of New Mexico Health Sciences Center, Albuquerque, New Mexico, United States of America; 3 inviCRO, LLC., Boston, Massachusetts, United States of America; 4 McAllister Heart Institute and Department of Pathology and Laboratory Medicine, University of North Carolina, Chapel Hill, North Carolina, United States of America; Texas A & M, Division of Cardiology, United States of America

## Abstract

Exposure to chronic hypoxia (CH) induces elevated pulmonary artery pressure/resistance, leading to an eventual maladaptive right ventricular hypertrophy (RVH). Muscle RING finger-1 (MuRF1) is a muscle-specific ubiquitin ligase that mediates myocyte atrophy and has been shown to play a role in left ventricular hypertrophy and altered cardiac bioenergetics in pressure overloaded hearts. However, little is known about the contribution of MuRF1 impacting RVH in the setting of CH. Therefore, we hypothesized that MuRF1 deletion would enhance RVH compared to their wild-type littermates, while cardiac-specific overexpression would reduce hypertrophy following CH-induced pulmonary hypertension. We assessed right ventricular systolic pressure (RVSP), right ventricle to left ventricle plus septal weight ratio (RV/LV+S) and hematocrit (Hct) following a 3-wk isobaric CH exposure. Additionally, we conducted dual-isotope SPECT/CT imaging with cardiac function agent ^201^Tl-chloride and cell death agent ^99m^Tc-annexin V. Predictably, CH induced pulmonary hypertension, measured by increased RVSP, RV/LV+S and Hct in WT mice compared to normoxic WT mice. Normoxic WT and MuRF1-null mice exhibited no significant differences in RVSP, RV/LV+S or Hct. CH-induced increases in RVSP were also similar between WT and MuRF1-null mice; however, RV/LV+S and Hct were significantly elevated in CH-exposed MuRF1-null mice compared to WT. In cardiac-specific MuRF1 overexpressing mice, RV/LV+S increased significantly due to CH exposure, even greater than in WT mice. This remodeling appeared eccentric, maladaptive and led to reduced systemic perfusion. In conclusion, these results are consistent with an atrophic role for MuRF1 regulating the magnitude of right ventricular hypertrophy following CH-induction of pulmonary hypertension.

## Introduction

Right ventricular hypertrophy (RVH) occurs in the setting of primary pulmonary arterial hypertension (PAH) and frequently precipitates mortality in this disease [1], with recent estimates of 44% of PAH patients dying from right ventricular failure or sudden cardiac death [2]. The manifestation of a dilated hypertrophic phenotype, characterized by a large RV volume and reduced stroke volume, correlates with an even worse prognosis [3]. RVH also contributes to mortality as a comorbid condition in a number of other syndromes, such as chronic obstructive pulmonary disease and scleroderma [4] [5]. Even without progressing to frank failure, progressive remodeling of the RV may generate a substrate for electrocardiographic abnormalities [6], which may contribute to the incidence of sudden cardiac death [7]. As such, improved understanding of molecular pathways that contribute to or modify RVH phenotypes may provide important clues towards novel therapies for PAH treatment.

Muscle RING finger 1 (MuRF1) is a ubiquitin ligase that regulates atrophy processes in striated muscle [8]–[10]. A critical component to the regulation of muscle mass is the turnover over and degradation of sarcomere proteins, which MuRF1 has been reported to ubiquitinate and target for proteasome-mediated degradation, including cardiac troponin I and beta-Myosin heavy chain [11], [12]. MuRF1 also interacts with a number of other sarcomere-associated proteins, including myosin light chain 2, titin, myotilin, and TnT, and may be responsible for the turnover of these and other yet to be identified proteins [13], [14]. However, the ability of MuRF1 to regulate signal transduction through its interaction with transcription factors may play a more prominent role in its regulation of cardiac hypertrophy. For example, in ischemia-reperfusion injury, MuRF1 regulates JNK-mediated apoptosis through its interaction, poly-ubiquitination, and degradation of phosphorylated c-Jun activated by reperfusion [15]. In pathological cardiac hypertrophy, MuRF1-/- mice undergo an exaggerated hypertrophy in vivo, suggesting an anti-hypertrophic activity of MuRF1 [8]. One possible mechanism by which MuRF1 exerts this activity is through its direct interaction with the transcription factor SRF, which it inhibits without affecting its protein level [8]. Using MuRF1 Tg mice with increased cardiac MuRF1 expression, it was shown that MuRF1 acts through the regulation of creatine kinase activity to alter cellular metabolism [10]. In models of cardiac atrophy and left ventricular cardiac hypertrophy reversal, MuRF1 is a critical mediator, as demonstrated in MuRF1-/- mice, resistant to both processes [9].

In contrast to the left ventricle, the right functions with low-pressure working conditions and a complex geometry different from the left ventricle. Despite the more passive role the RV may appear to play, it is important in the interdependence between left and right systolic and diastolic function. Pulmonary hypertension, for example, causes a leftward shift of the interventricular septum that can negatively affect LV function [16] [17]. Recent studies have investigated the role of the ubiquitin proteasome system in a right ventricular hypertrophy model [18]. After induction of PAH and the development of right ventricular hypertrophy, increased poly-ubiquitination and free ubiquitin was detected in the hearts [18]. Given MuRF1's presence in both the left and right ventricles and MuRF1's regulation of LV cardiomyocyte hypertrophy by multiple mechanisms, we hypothesized that MuRF1 would regulate right ventricular hypertrophy *in vivo*. To test this, we challenged MuRF1-/- and MuRF1 Tg+ mice to hypoxia-induced pulmonary hypertension to detect the role of MuRF1 in the compensatory mechanisms of a model of pulmonary artery hypertension.

## Methods

### Animal Model

Mice were obtained from colonies whose derivation has been previously described [8], [10] or from a commercial vendor (C57BL/6 mice from Taconic; Oxnard, CA) and shipped to the University of New Mexico. Following a week-long quarantine, mice were moved to a control chamber or normobaric hypoxia chamber, but maintained in standard shoebox cages with food and water available ad libitum throughout. The hypoxia chamber was set at 10.0% oxygen (partial pressure of oxygen roughly 65 mmHg in Albuquerque) and monitored both by the digital feedback-control system (Biospherix, Colorado) as well as by a secondary O_2_/CO_2_ monitor (O2Cap, OxiGraf, Inc.; Mountain View, CA). The hypoxia exposure lasted 3 weeks with twice-weekly cage changes and standard 12 h light∶dark cycle. Procedures were conducted under full isoflurane anesthesia to minimize or eliminate risk of pain and discomfort. All procedures were conducted with full approval by the University of New Mexico Institutional Animal Care and Use Committee and carried out in compliance with the National Institutes of Health Guide for the Care and Use of Laboratory Animals.

### Single-Photon Emission Computed Tomography

On the final day of hypoxia or normoxia exposures, mice were slowly injected via a bolus tail vein injection of approximately 0.75 mCi of ^201^TlCl in 0.9% NaCl, USP for ECG-gated SPECT/CT assessments of cardiac perfusion, volume and function. Imaging was conducted at approximately 30 minutes post injection as previously described [17]. As in this previous work, RV imaging in normoxic mice was occasionally sub-optimal due to a large signal from the left ventricle that overwhelmed our ability to discriminate the RV. For this reason, data for normoxic mice are frequently pooled to facilitate statistical comparisons. In hypoxic mice, RV hypertrophy was sufficient to mitigate this challenge.

Volume and radioactivity concentration were estimated from 3D regions-of-interest (ROIs), including left & right ventricle, left & right myocardium, entire heart, and fixed-volume lung and muscle sub-regions, generated using VivoQuant (inviCRO) image processing software. A flow quantification software FlowQuant (University of Ottawa, Heart Institute) was used to estimate cardiac ejection fraction.

### Right Ventricular Pressure, Hypertrophy, and Hematocrit

Immediately following the SPECT/CT assessments, mice were rapidly intubated and artificially ventilated with 2% isoflurane/balance O_2_. Under anesthesia, a thoracotomy was performed and a saline-filled catheter was inserted into the right ventricle. Stable pressure measurements were obtained for >30 seconds from which right ventricular systolic, mean, and diastolic pressures were obtained. Next, blood was rapidly withdrawn into capillary tubes and spun for hematocrit readings, while the heart was excised and carefully dissected into right ventricle and left ventricle + septum to obtain separate weights for derivation of RV/LVS ratios, as previously described [19].

### Quantitative Polymerase Chain Reaction

qPCR was conducted as previously described [20] [21] using a Roche 480 LightCycler instrument. Primers were obtained from a commercial vendor (Life Technologies) and shown in Table 1.

**Table 1:** Primers used for quantitative PCR.

Gene Name	Gene Symbol	Primer ID
TATA Box Protein	TBP	Mm00446971_m1
Muscle RING finger 1 (MuRF1)	Trim63	Mm01185221_m1
B-type Natriuretic Peptide (BNP)	Nppb	Mm01255770_g1
Beta Myosin Heavy Chain (bMHC)	Myh7b	Mm01249941_m1
Alpha Smooth Muscle Actin	Acta2	Mm01546133_m1
NADH-Ubiquinone Oxidoreductase Chain 2	MtND2	Mm04225288_s1

### Statistics

Considerations for pooling of data were given for normoxia and WT groups in all scenarios. Where either variation or means were statistically different, pooling was not conducted. However, for most endpoints, no differences were observed between WT KO and WT Tg+ in hypoxic groups or between any strain in normoxia. A clear exception to this was overall heart weights, where the Normoxic MuRF1-/- mice had evident cardiac enlargement compared to most other strains. All data were examined for assumptions of normal distribution. Data were routinely compared by 2-factor Analysis of Variance, considering the roles of hypoxia and genotype, and using a Bonferroni post-hoc test to explore specific group effects. Probability values of less than 0.05 were considered significant.

## Results

### Right ventricular MuRF1 mRNA expression decreases in response pulmonary hypertension

**Figure 1:**
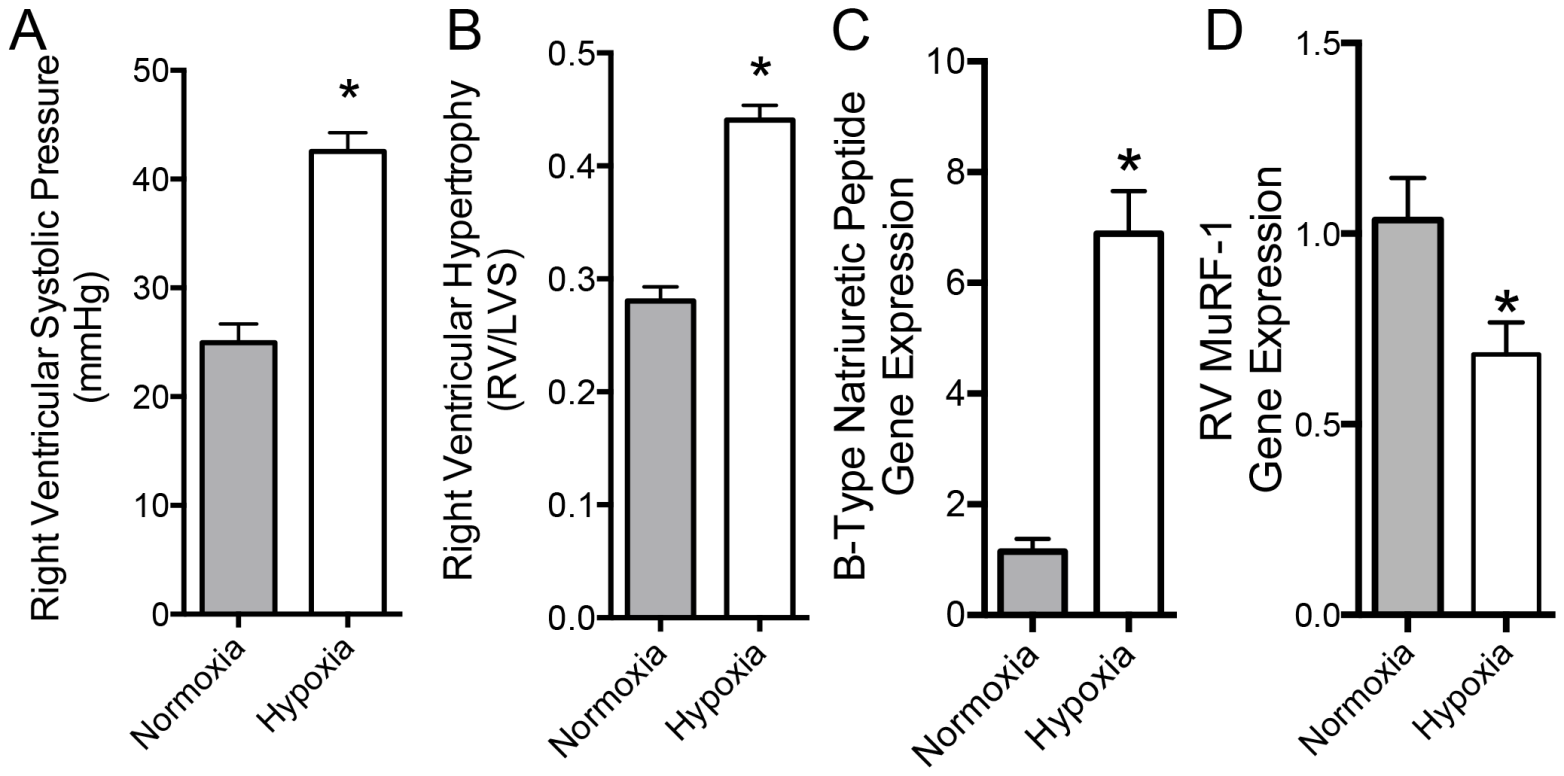
WT C57BL/6 mouse response to hypoxia in terms of right ventricular systolic pressure (A), right ventricular hypertrophy (B), right ventricular expression of B-type natriuretic peptide mRNA (C) and right MuRF1 mRNA (D). Asterisks denote significant difference from normoxic controls (p<0.05) by two-tailed t-test (N = 7-8 per group).

C57BL/6 mice challenged to chronic hypoxia exhibited increased right ventricular hypertrophy. Exposure to 10% O_2_ for 3 weeks resulted in significantly increased right ventricular systolic pressure, resulting in an increase in right ventricular mass measured by the Fulton index (RV/LVS; Fig. 1, A and B). BNP expression was significantly increased in the right ventricle, consistent with the hypertrophic response observed (Fig. 1C). The expression of the RV MuRF1 mRNA decreased compared to normoxic control mice (Fig. 1D).

### Chronic Hypoxia Increases Right Ventricular Pressure and Hematocrit

**Figure 2:**
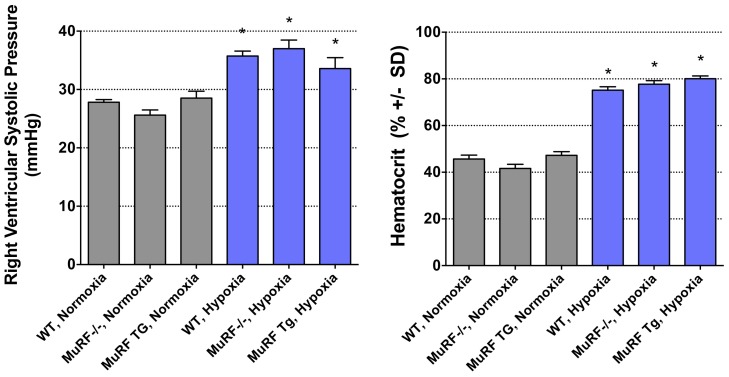
Hemodynamic and Hematologic Responses to Hypoxia in MuRF1 -/- and MuRF1 cardiac Tg+ mice. Chronic normobaric hypoxia (FiO_2_ = 0.10) led to predictable increase in right ventricular systolic pressure and hematocrit in WT and MuRF^-/-^ mice, as measured at 21 days (*left*). Asterisks denote significant difference from normoxia control groups (P<0.05; N = 5–11 per group). All strains exhibited increases in hematocrit (N = 5–11 per group, *p<0.001; *right*).

Both MuRF1-/-, MuRF1 Tg+, and their respective strain-matched wild type controls exhibited a statistically equivalent increase in RV systolic pressure 3 weeks of hypoxia (35.5–40.7 mm Hg) (Figure 2A, right). The systolic pressure in parallel normoxia treated mice did not differ between the pooled strain-matched WT controls, MuRF1 -/-, and MuRF1 Tg+ mice with mean values of 26.8, 25.6, and 28.5 mmHg, respectively (Figure 2A, left).

Hematocrit in normoxia-challenged controls was statistically similar across strains (Figure 2B, left). Hypoxia exposure induced a predictable increase in hematocrit, with values of 74.3, 77.7 and 80.1 for WT (pooled), MuRF1-/- and MuRF1 Tg+ strains, respectively, all significantly elevated over normoxic controls. Interestingly, in response to hypoxia, the MuRF1 Tg+ mice developed a significantly greater polycythemia than WT hypoxic mice (P<0.05, 2-way ANOVA with Bonferroni posthoc test). It should also be noted that we observed two lethalities in the MuRF Tg+ hypoxia group, one after two weeks and one just prior to imaging at 3 weeks. Lethality in the 10% O_2_ exposure is highly unusual in our experience.

### Weight Loss due to hypoxia is attenuated in MuRF1 -/- mice

**Figure 3:**
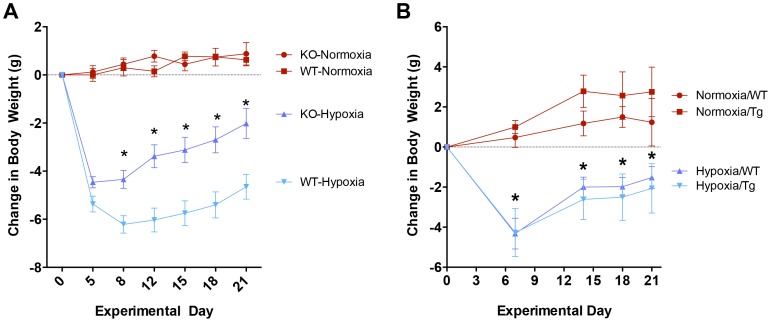
Body weight response to CH is evident in global *MuRF1 -/-* mice compared to cardiac-specific MuRF1 Tg^+^ mice. Longitudinal weight change profile during chronic hypoxia exposure illustrates global MuRF1 deletion attenuates weight loss with similar gain to WT *(A)*, whereas MuRF1 Tg^+^ mice have similar weight loss and gain compared to WT *(B)*. N = 5-6 per group, *p<0.05 from WT.

Normoxic control mice increased body weight over the 3 week experimental period, with no differences observed between the genotypes (Figure 3). WT, MuRF1-/- and MuRF1 Tg+ mice challenged with hypoxia in parallel underwent significant body weight, owing to reductions in food and water intake, of roughly 6 g over the first week (Figure 3A). MuRF -/- mice displayed a significant attenuation in this response compared to wildtype mice (Figure 3A), while MuRF1 Tg+ tracked consistently with their respective WT strain (Figure 3B). After one week, both MuRF1-/- and MuRF1 Tg+ mice undergoing hypoxia treatment stabilized body weight and consistently increased over the remaining 2 weeks.

### Right Ventricular Growth is Enhanced by MuRF1 Deletion, Unaltered by Overexpression

**Figure 4:**
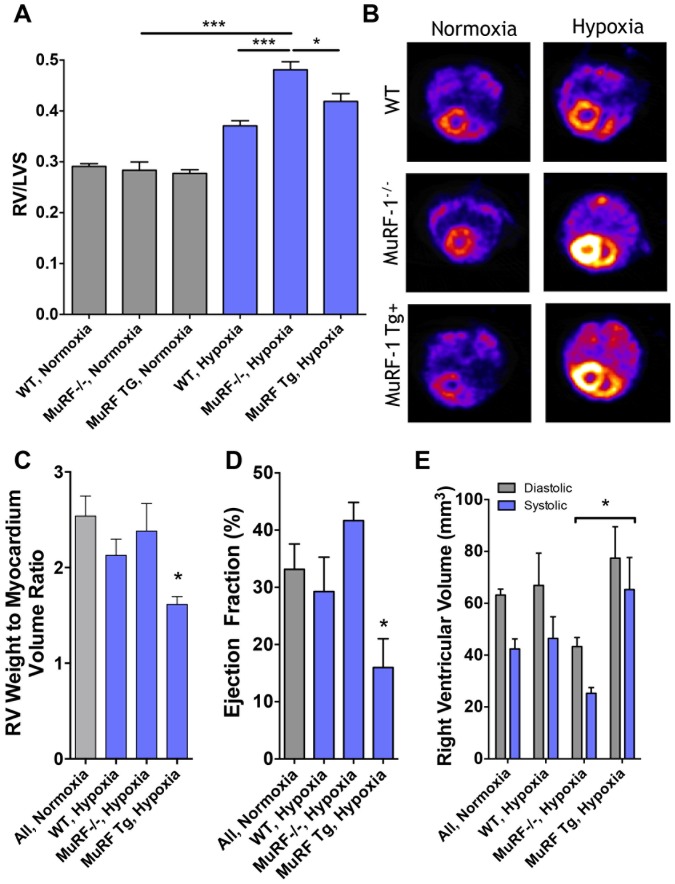
Hypoxia-induced right ventricular remodeling is enhanced by MuRF1 deletion, while cardiac-specific MuRF1 overexpression leads to a maladaptive dilated phenotype. (A) Chronic hypoxia induced predictable increases in RV/LVS, more so in the *MuRF1 -/-* mice than in WT. Interestingly, cardiac-specific overexpression of this atrophy-mediating ubiquitin ligase had no protective effect in terms of net hypertrophy of the right ventricle and in fact led to exacerbation of hypoxia-induced RVH relative to WT (*significantly greater than all control groups, *p*<0.01; **significantly greater than all control groups and WT hypoxia, *p*<0.01; ***significantly greater than all other groups, *p*<0.01). (B) Axial SPECT/CT cross-section images obtained at diastole reveal marked hypertrophy of the RV in hypoxia-exposed mice, with the appearance of dilation in the MuRF1 Tg+ mice. (C) Right ventricular weight to chamber volume ratio (D) ejection fraction and (E) RV systolic and diastolic volumes determined from ECG-gated SPECT/CT images are shown. As RV wall motion was difficult to image in normoxia mice, all genotypes are pooled. (N = 3-6 per group; *significantly lower than KO, Hypoxia mice, *p*<0.05).

Gravimetric and SPECT/CT assessment of the hearts from MuRF1 -/- and MuRF1 Tg+ mice challenged with hypoxia for 3 weeks demonstrated a significant right ventricular hypertrophy compared to all normoxic control groups (Figure 4). As predicted, hypoxia treatment induced a significant elevation in RV/LVS in the WT strains (0.37; pooled). MuRF1-/- mice after hypoxia treated exhibited an exaggerated cardiac hypertrophy, illustrated by a RV/LVS mean value of 0.48 that was significantly greater than both WT and MuRF1 Tg+ exposed to hypoxic conditions. Interestingly, mice with increased cardiac-specific MuRF1 expression and challenged with hypoxia for 3 weeks had increased right ventricular growth, rather than inhibited growth, as was expected. In fact, the MuRF1 Tg+ RV/LVS values (0.42) were significantly greater than WT, although significantly less than MuRF1-/- mice. SPECT/CT imaging revealed a pattern of RV growth in MuRF1 Tg+ mice that appeared more dilated than that of WT or MuRF1-/- mice (Figure 4B).

To quantify the degree of dilation observed in the SPECT/CT assessment, we compared the gravimetrically-determined mass of the RV free wall with the SPECT/CT-derived RV chamber volume, which showed a significant reduction in hypoxia exposed MuRF1 Tg+ mice relative to all other groups, confirming a dilated phenotype (Figure 4C). Ejection fractions were also determined on those subjects for whom adequate ECG-gated images were collected; notably normoxic mice had smaller RV mass compared to LV mass, which was a limitation in this study, necessitating pooling of data for all strains in the normoxia treatment (Figure 4D). Not only did MuRF1 Tg+ hearts appear dilated after 3 weeks of hypoxia treatment, they exhibited a significant decrease in RV ejection fraction compared to normoxic controls (P<0.05), in contrast to wild type and MuRF1-/- mice, which were generally unaffected (Figure 4D). Overall systolic and diastolic volumes in the MuRF1 Tg+ hearts also appeared dilated compared to MuRF-/- mice (Fig. 4E).

### MuRF1 and RV expression of pathologic cardiac hypertrophy markers in response to hypoxia

**Figure 5:**
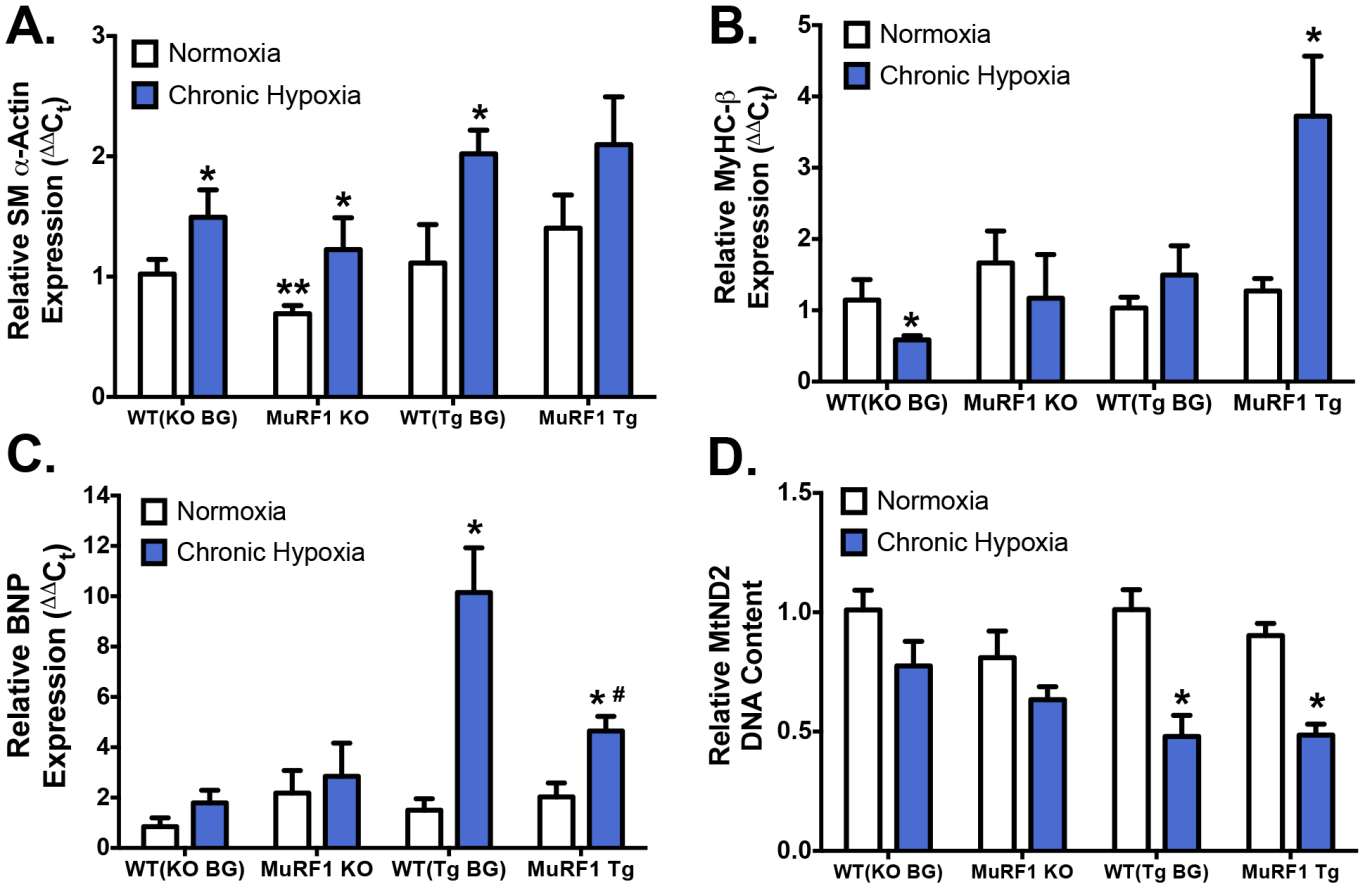
Differential Induction of Cardiac Hypertrophy/Failure Transcripts in MuRF^-/-^ & *MuRF1 Tg+* Mice Following CH. Effects of CH-induced PH on relative RV transcript expression of *(A)* SM a-actin, *(B)* MyHC-b, *(C)* BNP and *(D)* mtND2 DNA content from *MuRF1 -/-* and MuRF1 Tg+ mice. BNP, MyHC-b and SM a-actin expression normalized to TATA binding protein. MtND2 DNA content normalized to 18S DNA. N = 5 per group, *p<0.05 vs respective normoxic control; #p<0.05 vs chronic hypoxia Tg^+^; **p<0.05 vs respective WT.

The expression of mRNA normally found in the fetal heart, but not adult heart, were identified in mice challenged to hypoxia. Smooth muscle alpha-actin, myosin heavy chain beta, and BNP were measured in after 2 weeks of hypoxia challenge (Figure 5). Cardiac markers of pathologic hypertrophy demonstrated mixed outcomes related to the hypoxia and genetic factors. Hypoxia consistently induced RV expression of SM a-actin mRNA, with no major differences apparent among the strains (Fig. 5A). βMyHC mRNA did not show a substantial increase in most strains, despite the hypertrophic findings, but was significantly elevated in MuRF1 Tg+ mice. BNP mRNA was not significantly altered by chronic hypoxia in the MuRF1-/- mice or their genetic background strain, but both MuRF1 Tg+ and their background strain showed significant increases. Lastly, we assessed a marker of mitochondrial density, MtND2 DNA, which was significantly reduced by chronic hypoxia in both MuRF1 Tg+ mice and their background strain, but not in the MuRF1-/- mice or their background strain.

### Cardiac and Skeletal Muscle Perfusion

**Figure 6:**
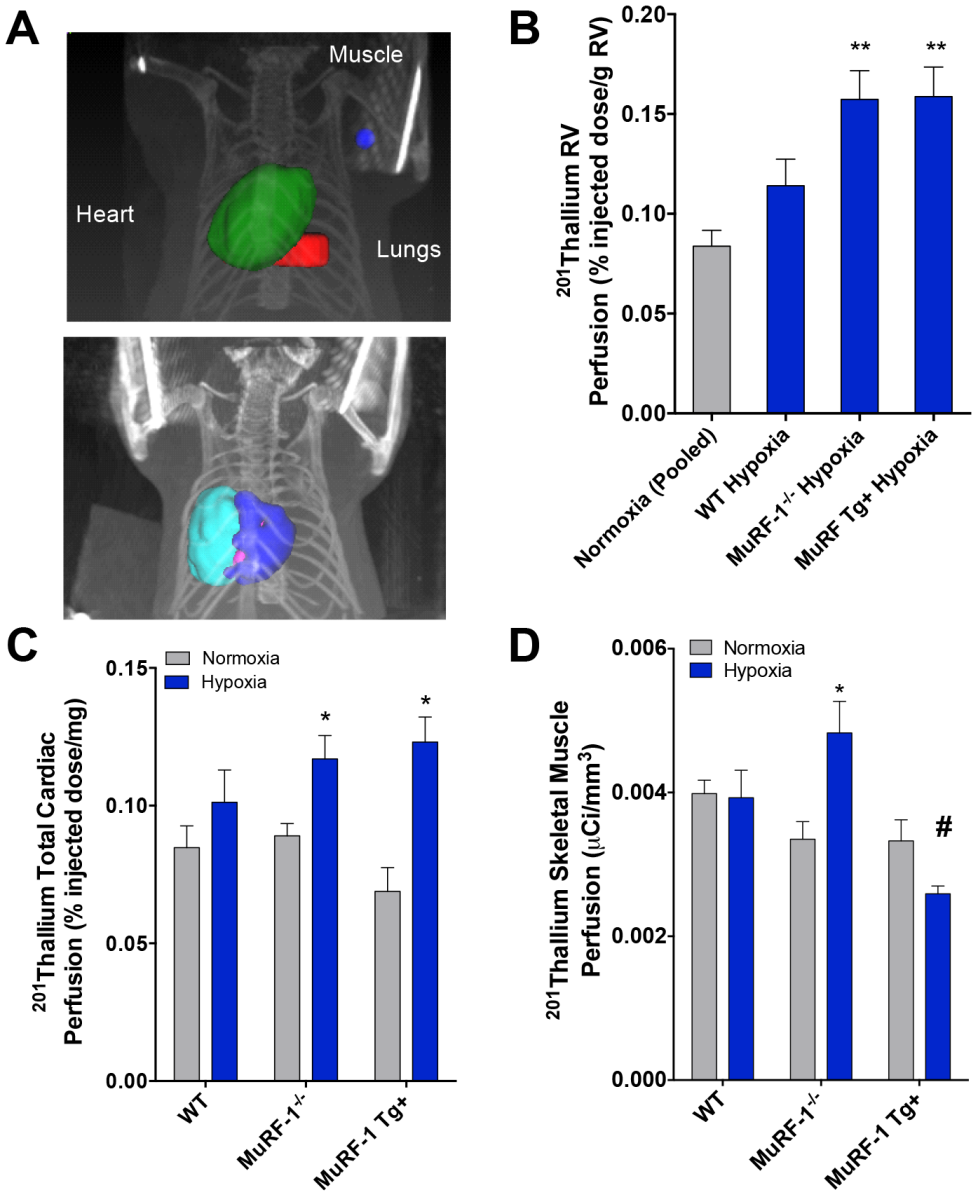
Relative perfusion of right ventricular myocardium, total heart, and skeletal muscle as a reference, as determined by SPECT/CT using ^201^TlCl uptake. Region of interest phantoms are depicted in (A). Hypoxia caused a significant increase in perfusion in the RV (B) of both *MuRF1 -/-* and *MuRF1 Tg+* mice relative to RV mass (N = 5–8 per group; ** denotes *p*<0.01 compared to normoxia mice by ANOVA). The RV perfusion was not consistently observable in controls, so results are pooled for all mouse models. The total heart uptake also showed an effect of hypoxia that was predominant in the *MuRF1 -/-* and *MuRF1 Tg+* mice relative to WT (C). Skeletal muscle perfusion (C) was elevated in hypoxia-exposed MuRF1 -/-mice but reduced in hypoxia-exposed *MuRF1 Tg+* mice. (B,C: N = 5–8 per group; * indicates *p*<0.05 compared to normoxic conditions for the same strain; † indicates P<0.05 compared to WT).

SPECT/CT imaging of ^201^TlCl was used to assess overall perfusion of cardiac tissue and a region of skeletal muscle in a small cohort of exposed mice (Fig. 6A). Again, because the normal RV is small in comparison to the LV, the normoxic RV was often difficult to reliably assess. For this reason, data for RV total perfusion in normoxic mice were pooled across strains in mice that could be assessed. RV perfusion was significantly elevated in hypoxia-exposed MuRF1-/- and MuRF1 Tg+ mice compared to normoxic controls (Fig. 6B). A modest, but non-significant perfusion increase was seen in hypoxia-exposed WT mice; a restricted group subanalysis of the hypoxia effect in WT mice by Student's *t*-test revealed a likely effect compared to normoxia-exposed mice (P = 0.035). When ^201^TlCl uptake for the whole heart was calculated, hypoxia as an independent treatment factor caused a significant increase in perfusion, most pronounced in the MuRF1-/- and MuRF1 Tg+ mice (Fig. 6C). However, when skeletal muscle perfusion was analyzed, MuRF1-/- and MuRF1 Tg+ mice trended in opposite directions, with greater skeletal muscle perfusion seen in MuRF1-/- mice and reduced perfusion in MuRF1 Tg+ hearts.

## Discussion

MuRF1 regulates atrophy of cardiac muscle through the degradation of structural and regulatory proteins [22]. In the setting of hypoxia-induced PAH, RV MuRF1 mRNA expression was downregulated, potentially to permit cellular hypertrophic responses. The deletion of MuRF1 expectedly led to exaggerated growth of the right ventricle relative to the left in response to hypoxia-induced PAH. However, cardiac-specific overexpression of MuRF1 did not have the opposite effect (*i.e.*, preventing growth), but rather led to a maladaptive phenotype associated with wall thinning, lower ejection fraction, and reduced perfusion. We speculate that selective overexpression of MuRF1 may enhance degradation and therefore reduce abundance of sarcomeric proteins, with minimal impact on growth-related signaling for other cellular components, thus allowing for enlargement of cells with reduced contractile force. On the other hand, inhibition (or deletion) of MuRF1 may permit an enhanced contractile phenotype. The short time frame (3 weeks) of the hypoxia model limits the conclusions as to the potential positive or negative implications of the enhanced growth in MuRF1-/- mice, but increased growth in the presence of adequately compensated perfusion suggests a beneficial adaptation.

Right ventricular failure is a common and defining outcome in many forms of pulmonary hypertension [4] [5]. Little research has been conducted on ride-sided cardiac hypertrophy and failure in terms of proteolysis and ubiquitin ligase involvement. However, a great deal is known about left-sided hypertrophy and failure and the role of MuRF1, specifically. In LV pathologic cardiac hypertrophy, MuRF1 inhibits pathologic cardiac hypertrophy in a transaortic constriction (TAC) model without affecting function [8], [10]. However, cardiac-specific overexpression of MuRF1 in the TAC mode led to eccentric remodeling similar to that seen in the present study with hypoxia-induced right ventricular remodeling [9]. MuRF1 regulates the creatine kinase activity in MuRF1 Tg+ hearts and increases susceptibility to TAC-induced heart failure, while doing little to inhibit cardiac hypertrophy. While much of this is consistent with the influence of MuRF1 overexpression on hypoxia-induced right ventricular changes, in the TAC model mitochondrial numbers were significantly increased in MuRF1 Tg+ mice, while we observed a reduction in cardiac MtDNA levels due to hypoxia that was unaffected by MuRF1.

We had previously investigated the roles of MuRF1 and a related ubiquitin ligase, atrogin-1 or MAFbx, in pulmonary vascular smooth muscle hypertrophy stemming from pulmonary hypertension [23]. In the monocrotaline (MCT) model, rat pulmonary arteries demonstrated significant reductions in atrogin-1 expression that tightly followed the timecourse of vascular hypertrophy and hemodynamic changes. We then used resveratrol to specifically upregulate atrogin-1, both in whole animal and vascular smooth muscle cell culture studies, which led to a reversal of the hypertrophic/hyperplastic phenotype. While off-target pleiotropic effects of resveratrol cannot be discounted, these findings motivated further inquiry into the role of ubiquitin ligases in aspects of PAH. Subsequent studies of MCT found atrogin-1 to be downregulated in the right ventricle, as well [21]. Interestingly, MuRF1 is not highly expressed in smooth muscle cells, despite its prominent role in skeletal and cardiac muscle growth.

Resveratrol was initially used in the MCT model due to our hypothesis that inducing a caloric restriction phenotype in myocytes would reduce growth pathways. In cardiac muscle this might reduce the size of the myocytes, while in smooth muscle it may also reduce hyperplasia. Pathways upregulated by caloric restriction, related to AMP kinase and sirtuin signaling, oppose IGF-1/Akt signaling in cardiomyocytes. AMPK, activated via caloric restriction or pharmacologically (AICAR), induces MuRF1 and atrogin-1 in cardiomyocytes [24]. Caloric restriction also maintains elevated levels of skeletal muscle MuRF1 and atrogin-1 [25]. These studies highlight a key involvement of MuRF1 in energy-saving benefits in scenarios of reduced energy bioavailability or increased metabolic demand, such as in the case of elevated pulmonary vascular resistance. However, for cardiomyocytes, activating MuRF1 and impeding the matched growth of contractile components compared to other cellular components may lead to maladaptive stretching of the myocytes and eccentric remodeling. Therapeutic interventions that antagonize MuRF-1, on the other hand, may permit the increased abundance of contractile proteins and enhance force generation, with the caveat that failure to remove aging sarcomeric components would soon lead to negative outcomes.

One interesting observation in this study was that MuRF1-/- mice were partially protected against the weight loss that occurred during the mid-to-late phases of the hypoxic exposure (Figure 3). The present hypoxia model incorporates a weight loss phase that is a combination of hypoxia and starvation, as the mice decrease activity and ingest less food and water in the first week of treatment. Weight loss in this model results in ∼6 g decrease in body weight in wild-type mice. Our findings suggest that MuRF-1 did not affect the initial magnitude of weight loss, which is consistent with the literature defining this initial period as a lack of food consumption. In comparable studies, hypoxia-induced weight loss is directly related to early deficits in food and water consumption; supplementing hypoxic rats with erythrocytes to offset reduced oxygen availability did not reduce the initial weight loss phase [26]. On the other hand, in the later phases of the hypoxia model the mice are chronically hypoxic and the absence of MuRF-1 led to a faster recovery of body weight, presumably muscular weight. Mice deficient in erythropoietin exhibit significant reductions in hematocrit and muscle oxygenation, which induces skeletal muscle proteolysis [27]. Rats in a 5-week hypobaric hypoxia model exhibited increased skeletal muscle expression of a related ubiquitin ligase, atrogin-1[28]. It may also be that hypoxia-related inactivity plays an important role, and it has been documented that MURF1 -/- mice are resistant to skeletal muscle atrophy in response to a denervation model [29].

It is unknown to what extent these factors (anorexia, hypoxia, inactivity) may also contribute to the cardiac muscle mass changes and MuRF1 activity beyond the obvious mechanical stress incurred by the hemodynamic changes in this PAH model. While more detailed histopathology and complementary functional assessments would be ideal, the data from this study strongly suggests that functionality of the whole heart is better preserved when MuRF1 is absent and impaired when it is overexpressed (Figure 6). In human heart failure, skeletal muscle undergoes a number of atrophic changes, including reduced Akt phosphorylation [30], mitochondrial density [31], and cross-bridge kinetics [32]. In MuRF1-/- mice, systemic perfusion was preserved, possibly even increased over controls, while the cardiac-specific MuRF1 overexpression led to reduced skeletal muscle perfusion, as assessed by SPECT/CT. The recovery of body weight in the MuRF1-/- mice may relate in part to this preserved cardiac function, but more detailed studies are needed to define this phenomenon.

One clear limitation to the present study is the lack of assessment of pulmonary vascular remodeling and resistance. Because we did not see clear differences in RVSP or HCT that would drive the pattern of RV/LVS phenotypes, we believe it is unlikely that a substantial load-dependent differential was incurred by the genetic manipulations in this study. MuRF1, unlike its relative ubiquitin ligase Atrogin-1 (paffet, that uterus study) [21] [33], does not appear to have an important role in smooth muscle regulation. Alternatively, one might consider a role for MuRF1 in response to hypoxia globally. This may in part explain the modest, significant increase in HCT in the hypoxic MuRF1 Tg+ mice compared to WT and MuRF1-/- mice, but postulating how a cardiac-specific overexpression of MuRF1 drives an increased erythropoeitic response would be challenging. Perhaps more compelling argument relates to the reduced perfusion in the failing heart of hypoxic MuRF1 Tg+ mice, which leads to reduced perfusion of kidneys and increased erythropoeitic signaling.

In summary, MuRF1 plays an important role in modulating the degree and quality of hypertrophic manifestations resulting from the hypoxia-induced PAH model. The overexpression of MuRF1 led to an eccentric and maladaptive hypertrophy, while deletion of MuRF1 permitted greater hypertrophy without a loss of function. While we foresee a limited and biphasic benefit, there may be a strategic therapeutic opportunity to antagonize MuRF1 to allow for improved growth of the RV to match elevated pulmonary arterial resistance. A better understanding of MuRF1 and overall proteasomal function in cardiac hypertrophic may enable interventions for modifying chronic hypertrophic remodeling towards a more benign outcome.
